# Broadening the spectrum of ivermectin: Its effect on *Trypanosoma cruzi* and related trypanosomatids

**DOI:** 10.3389/fcimb.2022.885268

**Published:** 2022-07-28

**Authors:** Laura Fraccaroli, María Daniela Ruiz, Virginia Gabriela Perdomo, Agustina Nicole Clausi, Darío Emmanuel Balcazar, Luciana Larocca, Carolina Carrillo

**Affiliations:** ^1^ Laboratorio de Biología Molecular y Bioquímica en Trypanosoma cruzi y otros agentes infecciosos, CONICET for Instituto de Ciencia y Tecnología (ICT) Milstein - Consejo Nacional de Investigaciones Científicas y Tecnológicas (CONICET), Buenos Aires, Argentina; ^2^ Área Parasitología, Microbiología, Facultad de Ciencias Bioquímicas y Farmacéuticas, Universidad Nacional de Rosario (UNR), Rosario, Argentina; ^3^ Centro de Estudios Parasitológicos y Vectores (CEPAVE), CONICET - Universidad Nacional de La Plata (UNLP), La Plata, Argentina

**Keywords:** Chagas disease, ivermectin, drug repurposing, trypanocidal drug, drug combination, *Trypanosoma cruzi*

## Abstract

Chagas disease is an endemic American parasitosis, caused by *Trypanosoma cruzi*. The current therapies, benznidazole (BZN) and nifurtimox (NFX), show limited efficacy and multiple side effects. Thus, there is a need to develop new trypanocidal strategies. Ivermectin (IVM) is a broad-spectrum antiparasitic drug with low human and veterinary toxicity with effects against *T. brucei* and *Leishmania* spp. Considering this and its relatively low cost, we evaluate IVM as a potential repurposed trypanocidal drug on *T. cruzi* and other trypanosomatids. We found that IVM affected, in a dose-dependent manner, the proliferation of *T. cruzi* epimastigotes as well as the amastigotes and trypomastigotes survival. The Selectivity Index for the amastigote stage with respect to Vero cells was 12. The IVM effect was also observed in *Phytomonas jma 066* and *Leishmania mexicana* proliferation but not in *Crithidia fasciculata*. On the epimastigote stage, the IVM effect was trypanostatic at 50 μM but trypanocidal at 100 μM. The assays of the drug combinations of IVM with BNZ or NFX showed mainly additive effects among combinations. *In silico* studies showed that classical structures belonging to glutamate-gated Cl channels, the most common IVM target, are absent in kinetoplastids. However, we found in the studied trypanosomatid genomes one copy for putative IMPα and IMPβ, potential targets for IVM. The putative IMPα genes (with 76% similarity) showed conserved Armadillo domains but lacked the canonical IMPβ binding sequence. These results allowed us to propose a novel molecular target in *T. cruzi* and suggest IVM as a good candidate for drug repurposing in the Chagas disease context.

## Introduction

Chagas disease, caused by the protozoan parasite *Trypanosoma cruzi*, is considered a neglected tropical disease (NTD) (World Health Organization, 2015). This originally American endemic affects at least 6 million people and has been globally spread with the recent migrations (Pérez-Molina and Molina, 2018), estimating that 75 million people are at risk of contracting the disease. The social and economic impact of this disease is significant, with 14,000 annual deaths and 40,000 new cases every year (Nunes et al., 2013).

In addition to the complexity of social, economic, and biomedical issues about Chagas disease, there are only two drugs accepted for its treatment: nifurtimox (NFX) and benznidazole (BZN). Both drugs, developed 50 years ago, show very variable parasitological cure rates depending on the disease stage and present several adverse effects (Nunes et al., 2013). Thus, there is a clear need to find new therapies against Chagas disease to provide better assistance and life quality to patients and to reduce the social and economic burden associated with contagion and chronicity. A cost- and time-saving strategy to discover new trypanocidal treatments is drug repurposing, finding new therapeutic indications for drugs that are already approved for its use in humans (Bellera et al., 2013).

Ivermectin (IVM) is a broad-spectrum antiparasitic drug, initially developed to combat parasitic worms in veterinary fields (Campbell et al., 1983). It is a mixture of semisynthetic macrocyclic lactones (~80% 22,23-dihydro-avermectin B1a and ~20% 22,23-dihydro-avermectin B1b) (Crump, 2017; Deng et al., 2019). Nowadays, IVM is used mainly as an oral medication in the primary treatment of onchocerciasis, lymphatic filariasis, and strongyloidiasis infections, all caused by different nematode species (Turner and Schaeffer, 1989; Laing et al., 2017). IVM also affects arthropod ectoparasites, being used in the treatment of scabies (Thomas et al., 2015), ticks (Sheele et al., 2014), and head lice (Devore and Schutze, 2015).

In WHO massive antiparasitic-drug administration programs, it was observed that the incidence of malaria seemed to decrease in pediatric groups treated with IVM (Alout et al., 2014; Chaccour and Rabinovich, 2019). Further studies showed that IVM has presented *in vitro* and *in vivo* antimalarial effects, arresting the cell cycle and inhibiting parasite development and survival (Mendes et al., 2017; de Carvalho et al., 2019). In regard to trypanosomatids, IVM showed effects on *Leishmania* spp. in animal models and patients’ therapy with cutaneous leishmaniasis (Opara and I.G, 2005; Kadir et al., 2009). IVM also showed promising results in the models of mice infected with *T. brucei* (Udensi and Fagbenro-Beyioku, 2012). Related to Chagas disease and IVM, few studies have been made, mainly centered in triatomine vectors (Dias et al., 2005; Dadé et al., 2014; Dadé et al., 2017). To our knowledge, the unique report of IVM in Chagas disease in humans was a descriptive work based on a survey conducted in a self-medicated population group living in an endemic area of Bolivia, in which IVM caused some improvements in their self-perception of Chagas disease symptoms (Forsyth, 2018).

Different mechanisms of action have been proposed to explain the IVM effect on diverse organisms. IVM potentiates the glutamate-gated Cl channel (GluCl), found in invertebrates (nematodes and arthropods) (Cully et al., 1994), resulting in the hyperpolarization of parasite neurons and muscles (Chen and Kubo, 2018). IVM also inhibits importin α/β (or “karyopherins”) (Wehbe et al., 2021), a heterodimer that transports proteins to the nucleus across the nuclear complex pore; this effect has been described in the asexual blood stages of *Plasmodium falciparum* (Panchal et al., 2014).

This evidence, together with its relative low cost, make IVM an interesting drug candidate to study for Chagas disease treatment. The aim of this work was to analyze the effect of IVM on different *T. cruzi* stages and other trypanosomatids’ proliferation and viability.

## Materials and methods

### Parasites and cell cultures

*T. cruzi* epimastigotes (Y, Dm28c/pLacZ, CL Brener, RA and Tulahuen 0 strains), *Phytomonas Jma 066* promastigotes, and *Crithidia fasciculata* choanomastigotes were cultured at 28°C in a Brain Heart Tryptose (BHT) medium supplemented with hemin (20 μg/ml) and *Leishmania mexicana mexicana* promastigotes (WHO strain), kindly provided by Dr. Cazzulo, were cultured at 28°C in SDM-79 (M199 media, Gibco, Grand Island, NY, USA). *T. cruzi* trypomastigotes and amastigotes were obtained from Dm28c/pLacZ epimastigotes expressing β-galactosidase, according to Alonso et al. (2021). Vero cells were maintained at 37°C and 5% CO_2_ atmosphere in Dulbecco's Modified Eagle Medium (DMEM) high glucose (Gibco) supplemented with 2 mM glutamine. All media were supplemented with streptomycin (100 μg/ml) and penicillin (100 IU/ml) and 2%–10% heat-inactivated fetal bovine serum (FBS; Natocor, Córdoba, Argentina) as indicated.

### Compounds and drugs

The stock solutions of the drugs (50 mM) were prepared in dimethyl sulfoxide (DMSO) for IVM (MW: 875.1, kindly provided by Pablo Cassará Laboratory), NFX (MW: 287.3, Sigma) and BZN (MW: 260.25, Sigma, Saint Louis, MO, USA). The stock solutions were diluted in culture media to obtain the working concentrations at the moment of the assay.

### Effect of ivermectin on *T. cruzi* and related trypanosomatids in culture

The effect of IVM was tested after 72 h of culture, by counting the number of mobile parasites in a hemocytometer chamber, by measuring optical density (OD) at 630 nm and by the Thiazolyl Blue Tetrazolium Bromide (MTT) assay (Frank and Pace, 1998), as indicated. For this last methodology, treated cells were incubated for 3:30 h at 28°C in the absence of light. The formed formazan was resuspended in DMSO. The absorbance was read at 570 nm using an ELISA plate reader (Cambridge Technology, Lexington, MA, USA) using wells with only media and reagents as reaction blank.

For Dm28c/pLacZ epimastigote, trypomastigote, and amastigote cultures, the proliferation was measured indirectly by the cytoplasmic β-galactosidase activity. Briefly, after the treatment with IVM, parasites were lysed and incubated with the enzyme substrate, chlorophenol red β-D-galactopyranoside. The colorimetric reaction was measured at 570 nm (Alonso et al., 2021).

Under same culture conditions, DMSO (at the highest concentration used) and NFX or BZN were used as negative and positive controls, respectively.

### Mammalian cytotoxic activity

Cytotoxicity was *in vitro* tested on Vero cells treated with increasing concentrations of IVM diluted in DMEM (up to 15 µM). The plate was then incubated for 24 h at 37°C with 5% CO_2_, and the MTT assay was performed as previously explained (see *Effect of ivermectin on T. cruzi and related trypanosomatids in cultur*e).

### Cell proliferation recovery assay

Recovery assays were performed as Kessler et al. (2013). Briefly, *T. cruzi* epimastigotes were preincubated with IVM at 50 and 100 μM (four and eight times the calculated IVM EC50_72h_, respectively), during different periods of time (from 30 min to 3 h). Then, parasites were washed with sterile PBS, transferred to a drug-free BHT 10% FBS medium, and incubated at 28°C. Growth recovery was monitored at days 4 and 8 by cell density in a hemocytometer chamber and OD at 630 nm.

### Drug combination study

To evaluate the effect of interaction between IVM and BZN or NFX on *T. cruzi* epimastigotes, a drug combination matrix was designed to test both drugs simultaneously or in a sequential manner. For simultaneous assays, the serial dilutions of IVM (0–50 μM) were placed in the columns of a 96-well plate and increasing concentrations of the second drug (BNZ or NFX) were added in each row, and then, parasites were seeded. For sequential assays, epimastigotes were first preincubated with IVM, NFX, or BZN in the corresponding matrix concentration; after 1 h, parasites were washed and seeded in a 96-well plate and the second drug was added following the combination matrix. The final concentration of DMSO was at most 0.5%. Treated cultures, blank samples (only BHT and drugs in their maximal concentration), and negative control (parasites in 0.5% DMSO) were incubated at 28˚C for 72 h, and parasite proliferation was measured by OD at 630 nm. Data were analyzed using the free software Combenefit (v. 2.021, Cambridge University, Cambridge, UK) (Di Veroli et al., 2016). Relative epimastigote proliferation obtained experimentally was compared against the predicted values according to *Bliss Independence* or *Loewe additivity* models. The synergy score calculated by the software is a positive value for a synergic effect, 0 for an additive effect (when drugs have no interaction), and negative for an antagonic effect. The results obtained were depicted in a synergy distribution plot where effects are indicated by a scale of colors and numbers, and statistical differences are marked with an asterisk.

### *In silico* analysis

In order to postulate a possible mechanism of action of IVM in *T. cruzi*, an *in silico* study was performed, supported with bibliographic data. To search for orthologous protein sequences of importin α (IMPα) and importin β (IMPβ) in the trypanosomatids of interest (*T. cruzi, L. exicana, C. fasciculata*, and *Phytomonas* spp.) and other close-related species, TritrypDB (*Tritrypdb.org*), Uniprot (www.uniprot.org/), and National Center for Biotechnology Information (NCBI) databases were used. Sequence alignments and analysis were performed with ClustalOmega (www.ebi.ac.uk/Tools/msa/clustalo/), GeneDoc 2.7 (www.psc.edu/biomed/genedoc), and Jalview 2.11.1.2 (www.jalview.org). Protein domains were identified using PFAM (Mistry et al., 2021) and SMART (Letunic et al., 2021) online tools, both developed by EMBL-EBI.

### Statistical analysis

All assays were independently performed in triplicate and data expressed as mean ± SEM. The significance of differences was evaluated with one-way analysis of variance (ANOVA) with *post-hoc* analysis using Dunnet’s test. A p <0.05 was considered significant. To calculate the EC50_24/72h_ of the drug, normalized proliferation or viability values were plotted against the Log of drug concentration (μM) and fitted to a sigmoidal curve determined by a non-linear regression. The selectivity index (SI) was calculated as CC50_Vero cells_/EC50_amastigotes_. Tests were performed using the GraphPad prism version 5.00 for Windows (GraphPad Software, La Jolla, CA, USA, www.graphpad.com).

## Results

### Effect and specificity of ivermectin on *T. cruzi*, other related trypanosomatids and host cells

The drug effect was evaluated, as a first approach, by following epimastigote cultures during 8 days with/without increasing concentrations of IVM, NFX, and BNZ. IVM affected *T. cruzi* epimastigote proliferation in a dose-dependent manner with an EC50_72h_ of 12.5 μM for the Y strain (**Supplementary Figure 1**) and 5.3 uM for the Dm28c strain. Other strains of *T. cruzi* (CL Brener, RA and Tulahuen 0) showed EC50_72h_ values ranging from 8.1 to 9.2 μM (data not shown). These are encouraging values as they are found between those obtained for us for the reference drugs (**Table 1**) and in bibliography (Luna et al., 2009; Alonso et al., 2021).

**Table 1:** EC50 values (μM) of ivermectin, nifurtimox, and benznidazole were determined in *T. cruzi* (Y strain-DTU II epimastigotes; Dm28c—Discreet Typing Units (DTU) I epimastigotes, trypomastigotes, and intracellular amastigotes), *C. fasciculata* choanomastigotes, and *L. mexicana* and *Phytomonas jma 066* promastigotes.

Organism	Life cycle form	µM	IVM	NFX	BZN
***Trypanosoma cruzi*** ***Y strain***	***Epimastigote***	EC50_72h_	12.5 (9.4–16.8)	2.1 (1.2–3.5)	22.2 (16.3–30.4)
***Trypanosoma cruzi*** ***Dm28c strain***	***Epimastigote***	EC50_72h_	5.3 (3.8–7.3)	0.9 (0.6–1.5)	3.8 (1.8–8.0)
	***Trypomastigote***	EC50_24h_	10.4 (7.0–15.5)	16.1 (7.6–33.8)	35.3 (11.3–110.1)
	***Amastigote***	EC50_24h_	0.3 (0.2–0.6)	1.4 (0.8–2.2)	0.8 (0.4–1.7)
***Leishmania mexicana***	***Promastigote***	EC50_72h_	9.7 (6.9–13.6)	3.8 (2.8–5)	7.9 (5.8–10.6)
***Phytomonas jma 066***	***Promastigote***	EC50_72h_	5.7 (4.6–7.5)	1.8 (1.1–2.7)	28.5 (20.7–39.1)
***Crithidia fasciculata***	***Choanomastigote***	EC50_72h_	ne	3.4 (1.9–5.8)	ne

ne, no effect observed.

The Dm28c strain expressing β-galactosidase, a quick and reproducible drug screening method, was used to evaluate the sensitivity of the trypomastigote and intracellular amastigote forms to IVM. Trypomastigotes showed an EC50_24h_ of 10.4 μM, while intracellular amastigotes showed more sensitivity with an EC50_24h_ of 0.3 μM (**Table 1**).

The IVM effect was also evaluated in trypanosomatid species related to *T. cruzi*; while it did not show effects on *C. fasciculata* (an insect trypanosomatid) under the evaluated concentrations (up to 200 μM), this drug affected the proliferation of *Phytomonas jma 066* (a plant parasite) and *L. mexicana* (a human disease–causing parasite) with an EC50_72h_ of 5.7 μM and 11.7 μM, respectively (**Table 1**). These results show that IVM has a specific broad range of effects on the different species of trypanosomatids as it has for other human pathogens.

The IVM cytotoxic effect at 24 h in Vero cells (CC50_24h_) was 3.6 µM (2.8–4.6) that, related to the EC50_24h_ in amastigotes (both assays performed in similar conditions), results in a selectivity index (SI) of 12, being an SI value ≥10 commonly assumed as a promissory basal (Katsuno et al., 2015).

### Effect of ivermectin in cell recovery assays in *T. cruzi* epimastigotes

**Figure 1:**
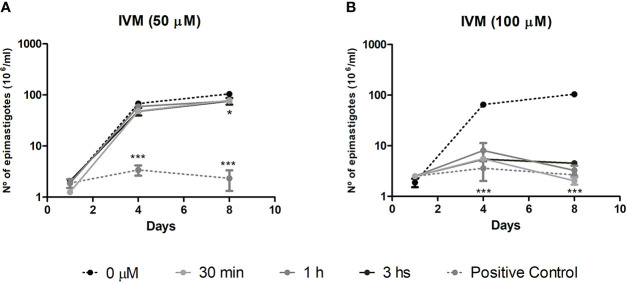
Cell proliferation recovery assay to test the effects of ivermectin (IVM) when exposed for short periods of time. *T. cruzi* epimastigotes were preincubated with **(A)** IVM 50 μM (4xEC50_72h_) or **(B)** IVM 100 μM (8xEC50_72h_) for 30 min, 1 or 3 h and then transferred to a drug-free medium for 8 days of culture. Untreated control: 0 μM IVM (0.5% dimethyl sulfoxide); positive control: 50 or 100 μM IVM during the 8 days of culture. Results are expressed as the number of epimastigotes (10^6^)/ml, counted in a hemocytometer chamber. ***p<0.001, *p<0.05, ANOVA, Dunnett test.

To evaluate whether the effect of IVM was trypanostatic or trypanocidal, cell recovery assays were performed. The effect of a drug is considered trypanostatic when parasite proliferation recovers in a drug-free medium after a short exposure to high drug concentrations and trypanocidal when it has irreversible effects on parasite proliferation (Mosquillo et al., 2018). At 50 μM IVM (4× EC50_72h_), we observed that epimastigote proliferation was recovered by removing the drug after incubation for up to 1 h, indicating a trypanostatic effect, followed by a significant slight proliferation decrease effect after 3 h treatment (**Figure 1A**). The assays performed with 100 μM IVM (8× EC50_72h_) showed an irreversible effect of the drug on parasite proliferation, independently of the exposure time, indicating a trypanocidal effect at this concentration (**Figure 1B**). According to these results, IVM treatment during short periods of time can be trypanostatic or trypanocidal depending on the concentration used.

### Effect of ivermectin in combination with benznidazole and nifurtimox

**Figure 2:**
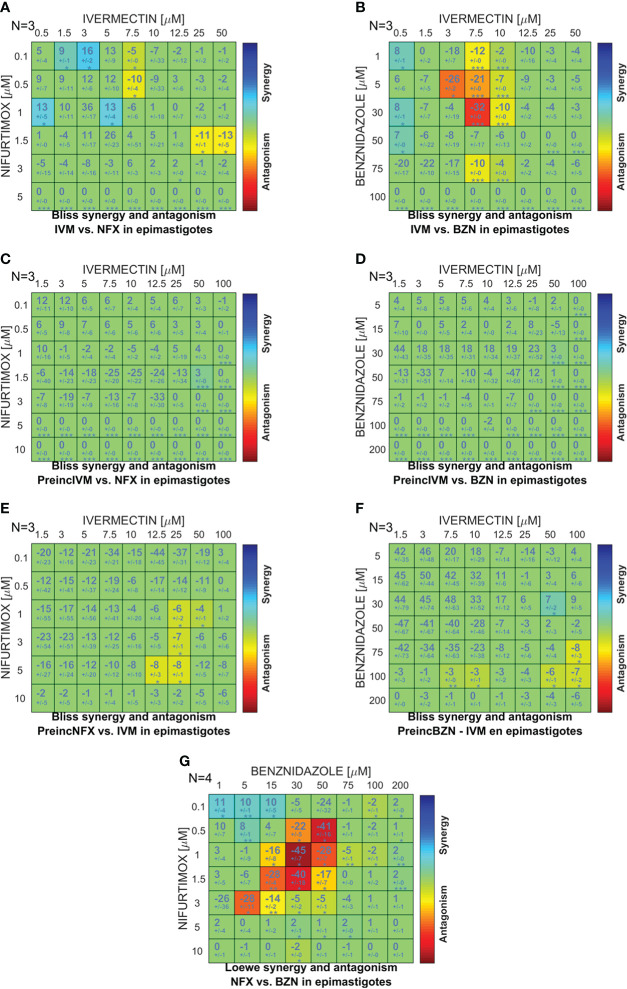
Comparison between the *in vitro* and predicted effects of drug combinations on *T. cruzi* epimastigote proliferation. A combination matrix was designed with IVM (0–50 µM) and benznidazole (BZN; 0–100 µM) or nifurtimox (NFX; 0–5 µM), and the drug combination effect was evaluated on the culture proliferation of epimastigotes at day 4. The results obtained in the *in vitro* culture assay were compared against the synergy scores predicted by applying the *Bliss independence* model for: **(A)** IVM-NFX and **(B)** IVM-BZN combined simultaneously; **(C)** IVM-NFX and **(D)** IVM-BZN with the preincubation of IVM for 1 h; **(E)** NFX-IVM and **(F)** BZN-IVM with preincubation of the trypanocidal drug for 1 h; and **(G)** by applying *Loewe additivity* model for the combination of NFX-BZN simultaneously. The scale of numbers and colors represents the type of effect obtained according to the comparison between *in vitro* and model-predicted results; the scale spans from statistically significant synergistic interaction (positive values—blue color, p<0.01) to statistically significant antagonism (negative values—red color, p<0.01).

To study the effect of the simultaneous or sequential combination of IVM with BZN or NFX, epimastigotes were cultured in combination matrices and the obtained data were analyzed with the free software Combenefit (Di Veroli et al., 2016), in which the null hypothesis of no synergy needs to be defined in order to assess the degree of the effect. In the case of IVM+BZN and IVM+NFX, the hypothesis was that these drugs have different mechanisms of action, taking the *Bliss independence* model as the best approach (Seguel et al., 2016; Tang, 2017). When the drugs were combined simultaneously, synergy distribution heatmaps-obtained from the comparison of experimental data with model-predicted values-showed an additive behavior in most of the concentrations. However, there were antagonistic effects at some combinations with middle and high concentrations of IVM (**Figures 2A, B**), and, on the other hand, some synergistic points at low concentrations of IVM-NFX were observed. In sequential assays, when IVM was preincubated for 1 h and then NFX or BZN was added, we only observed additive effects (**Figures 2C, D**), but when the preincubation was done with NFX or BZN, aside from additive effects, a slight antagonistic behavior at high concentrations of drugs was also observed (**Figures 2E, F**). Finally, we performed a control combination with NFX-BZN using the *Loewe additivity* model, based on the hypothesis that both drugs have common mechanisms of action (Tang, 2017), and the observed effects of combinations were mainly additive or antagonistic (**Figure 2G**).

### Bioinformatic analysis of potential drug targets and mechanism of action of ivermectin in *T. cruzi*

In order to find a possible mechanism of action of IVM on *T. cruzi*, different known targets of the drug in other organisms were studied. A first search in *TritrypsDB*, performed for ligand-activated chloride channels, did not show any positive coincidence in the genome of *T. cruzi*. To verify these results, an extensive bibliographic search was performed, finding well-established phylogenetic relations between homologous genes encoding for a GluCl along the kingdoms of life (Rendon et al., 2011). Other authors, applying three independent approaches (Psi-BLAST, HMMer, and Interpro, all based on hidden Markov models), found genetic sequences that codify for pentameric ligand–gated ion channel structures from Cys-loop receptors in a wide array of protists (Jaiteh et al., 2016) that did not include kinetoplastid genomes. Then, we conclude that IVM would be acting through another mechanism in *T. cruzi*.

Another studied mechanism of IVM action is the inhibition of nuclear transport by dissociating the performed importin α/β1 (IMPα/β1) heterodimer as well as by preventing its formation (Yang et al., 2020). At the moment, there is a unique report about IMPα in *T. cruzi* (Canela-Pérez et al., 2020). In order to deepen this work, we found putative sequences of IMPα in *T. cruzi* (Gene DB: TcCLB.509965.110), *L. mexicana* (Gene DB: LmxM.29.1120), *C. fasciculata* (Gene DB: CFAC1_260031300), and *Phytomonas* spp. (Protein DB: W6KHG4). TcIMPα is present in the CL Brener strain, Esmeraldo-like and non-Esmeraldo-like haplotypes, in chromosome 32; its length is 1,602 bp, and the predicted protein has 533 aa. We also found one copy of IMPβ (Gene DB: TcCLB.504105.150). Only one variant of each protein is reported in trypanosomatids, unlike mammals in which there exists a family of IMPα and IMPβ (in humans, seven IMPα and more than 20 IMPβ), showing the relevance of the *T. cruzi* heterodimer as a potential druggable target (Wagstaff et al., 2012).

A structural characterization of the IMPα sequences was made by the alignment of these proteins with those already characterized in *Mus musculus* (UniProtKB DB: P52293), *Homo sapiens* (UniProtKB DB: P52292), and *P. falciparum* (UniProtKB DB: Q7KAV0) (**Supplementary Figure 2**). Each sequence was analyzed with Pfam and SMART (EMBL) in order to determine domains and relative positions. All proteins had seven-to-nine Armadillo domains (ARM), separated by a conserved glycine (Canela-Pérez et al., 2020), with each ARM being composed of three hydrophobic α-helices (Cingolani et al., 1999). These repeated ARM domains end with an atypical ARM repeat (ARM9), which could be the linker with CAS exportin, and the IBB domain (Importinβ-binding domain) is found in the first 100 amino acids (N-terminal) in mammals as *H. sapiens* and *M. musculus*. In trypanosomatids, ARM repeats appear after the first 100-110 aa but there is not a typical recognizable IBB, which could be related to the presence of an IBB domain with particular characteristics (**Supplementary Figure 2**).

## Discussion

Drug repositioning is a widely used strategy to find new treatments for Chagas disease; several of the latest clinical trials were performed with repurposed drugs as posaconazole and fosravuconazole (Field et al., 2017). With IVM being a secure broad-spectrum antiparasitic drug used in human diseases, this drug would be an attractive repurposing candidate to study. Curiously, there are few reports that tangentially relate it with some Chagas disease aspects. Here, we present the first approach to study the effects of IVM on *T. cruzi* as a potential drug for Chagas disease.

In this report, we showed that IVM affected in a dose-dependent manner the proliferation of *T. cruzi* epimastigotes-a simple model to study trypanocidal drug effects-(EC50_72h_= 5.3–12.5 μM in the different strains evaluated) as well as amastigote (EC50_24h_=0.3 μM) and trypomastigote survival (EC50_24h_=10.4 μM). As BZN and NFX, IVM resulted to be more effective against amastigote stage, but with lower EC50 values (Luna et al., 2009), arousing interest as a potential trypanocidal drug. Moreover, the SI of IVM obtained for *T. cruzi* amastigotes was 12 in agreement with the drug selection criteria for infectious diseases that establish an SI value ≥10 as a promissory limit (Katsuno et al., 2015). On the other hand, the SI of NFX and BZN reported in bibliography are very variable, with values ranging from 1.5 to over 200 according to the strain and the cell line used (González et al., 2020). Reinforcing the idea of IVM as possible therapy against *T. cruzi* in humans, published pharmacokinetic data have shown a safety profile for the human use of the oral administration of IVM without adverse side effects, reaching plasmatic concentrations in the range of the EC50 values found in this work (Guzzo et al., 2002).

IVM also inhibited the *in vitro* proliferation of *Phytomonas jma 066* and *L. mexicana*, as previously shown in *L. tropica* (Kadir et al., 2009), while *C. fasciculata* choanomastigotes resulted to be highly tolerant to IVM and BZN. Throughout our results, IVM has shown effects on a broad but specific range of trypanosomatids as it has for other human pathogens.

We found that the trypanocidal capacity of IVM depends on its concentration (8xEC50_72h_) but not on the exposure time. An irreversible effect was also observed on *P. falciparum* at 25-µM IVM during 24 h of exposure (Panchal et al., 2014). Different mechanisms of cellular death (as necrosis or autophagy) could be triggered in *T. cruzi* parasites in response to drugs (Kessler et al., 2013). Moreover, the same compound can trigger different-reversible or irreversible-mechanisms according to the concentration and duration of the *stimuli*. Additionally, when the underlying mechanism of the drug is trypanostatic, parasitemia could show a relapse after antitrypanosomal chemotherapy (Ercoli and Iudice, 1980). This variable behavior of a compound is an important issue for therapy design.

In the last few years, the investigation of drug combinations has become an important strategy to combat drug resistance and reduce treatment courses in NTDs. Here, different drug combination regimens were tested *in vitro* on the epimastigote culture, finding that a simultaneous combination of IVM-BZN and IVM-NFX showed mainly additive effects; however, some combinations showed significant antagonistic or synergic effects. These effects could be explained by different described mechanisms of IVM action, including an antagonism by increasing detoxification processes induced by this drug that avoid the effects of NFX or BZN (Lespine et al., 2007; Ménez et al., 2012; Nodari et al., 2020) or a synergism by inhibiting the activity of P-gp, a member of the superfamily of transporters ABC (ATP-binding cassette), that enhances the cytotoxicity of other drugs (Furusawa et al., 2000; Landfear, 2019). As mentioned by other authors, the *in vitro* assays about the interaction between two drugs are usually the first-step studies, although the observed results do not necessarily reflect the *in vivo* effects; pharmacokinetic and host factors (as the immune response) may lead to different outcomes due to the modification of the effective exposure of the parasites to the drugs (Vincent et al., 2012).

The IMP*α* and IMPβ heterodimer complex, one of the IVM molecular targets, was searched in trypanosomatid genomes. One copy of each gene was detected in *T. cruzi*, *L. mexicana*, *Phytomonas* spp., and *C. fasciculata*, in agreement with previous studies (Canela-Pérez et al., 2020). Through multiple sequence alignment and structural analysis, we identified ARM domains (between seven and nine, according to the organism) while the typical IBB domain was not recognizable in these trypanosomatids.

The IMP*α* ARM repeat domain is relevant for the interaction with cargo proteins *via* a nuclear localization sequence (NLS), a short amino acid motif, present in cargo proteins (Yang et al., 2020). The evidence of conserved ARM domains in trypanosomatids and the *in vitro* studies showing their capacity of recognizing and binding to the NLS of *T. cruzi* (Canela-Pérez et al., 2019) suggest that this conserved mechanism of intracellular trafficking is worthy to be studied (Canela-Pérez et al., 2020).

The IBB domain, in N-terminus of IMP*α*, has three clusters of basic amino acid residues, and it is essential for binding to IMPβ and for efficient nuclear entry (Cingolani et al., 1999); in addition, the IBB domain can bind to the NLS-binding site of IMP*α*, leading to the autoinhibition of intracellular transport (Dey and Patankar, 2018). However, most protozoans lack a typical IBB, showing an early divergence (Dey and Patankar, 2018; Mayol et al., 2020). For example, *P. falciparum*, *T. gondii*, and *G. lamblia* do not present the three conserved clusters of basic amino acids in this region, and particularly, *P. falciparum* has shown to have a reduced autoinhibition mechanism of nuclear transport (Dey and Patankar, 2018; Mayol et al., 2020).

IVM is nowadays considered a specific inhibitor of the classical transport pathway of nuclear proteins. Although little is known about nuclear transport in *T. cruzi*, IVM has been associated with the inhibition of nuclear transport by *Tc*IMP*α*, showing that 25 μM IVM impaired the nuclear transport of an RNA polymerase of *T. cruzi* and 250 μM IVM has an inhibitory effect on the binding ability of *Tc*IMP*α* to an NLS peptide (Canela-Pérez et al., 2018 and Canela-Pérez et al., 2020). The accumulating evidence about importins makes them an attractive potential druggable target to continue studying. Finally, the study of the mechanisms of action of IVM on *T. cruzi* and other trypanosomatids is relevant to understand the currently unknown aspects of the parasite biochemistry and to discover novel molecular targets with potential for trypanosomatid therapy.

## Data availability statement

The raw data supporting the conclusions of this article will be made available by the authors, without undue reservation.

## Author contributions

LF, MR, and CC have worked in the study design, writing, and result discussion; LF and MR have made most of the experimental work. VP has conducted the experiments in the infective stages of *T. cruzi*. AC and LL have contributed with proliferation assays and DEB with bioinformatic work and analysis. LF and CC coordinated the investigation. LF and MR contributed equally, sharing the first authorship. All authors have contributed and given approval to the final version of the manuscript.

## Funding

The present work was supported by ICT Milstein - CONICET and The National Agency of Scientific and Technological Promotion (funds PICT 2015-0962 and PICT 2018-1124).

## Acknowledgments

The authors would like to thank CONICET, the Argentine National Council responsible for RRHH, ANPCyT, the Argentine National Agency that funded this work, and Laboratorio Pablo Cassará for donating the ivermectin used in this work.

## Conflict of interest

The authors declare that the research was conducted in the absence of any commercial or financial relationships that could be construed as a potential conflict of interest.

## Publisher’s note

All claims expressed in this article are solely those of the authors and do not necessarily represent those of their affiliated organizations, or those of the publisher, the editors and the reviewers. Any product that may be evaluated in this article, or claim that may be made by its manufacturer, is not guaranteed or endorsed by the publisher.
