# Supplementary material for: Broadening the spectrum of ivermectin: Its effect on Trypanosoma cruzi and related trypanosomatids
Source: Front Cell Infect Microbiol. 2022 Jul 28;12:885268. doi: 10.3389/fcimb.2022.885268 (PMC9366347; doi:10.3389/fcimb.2022.885268)
Supplement: Supplementary file 1 [file DataSheet_1.docx]

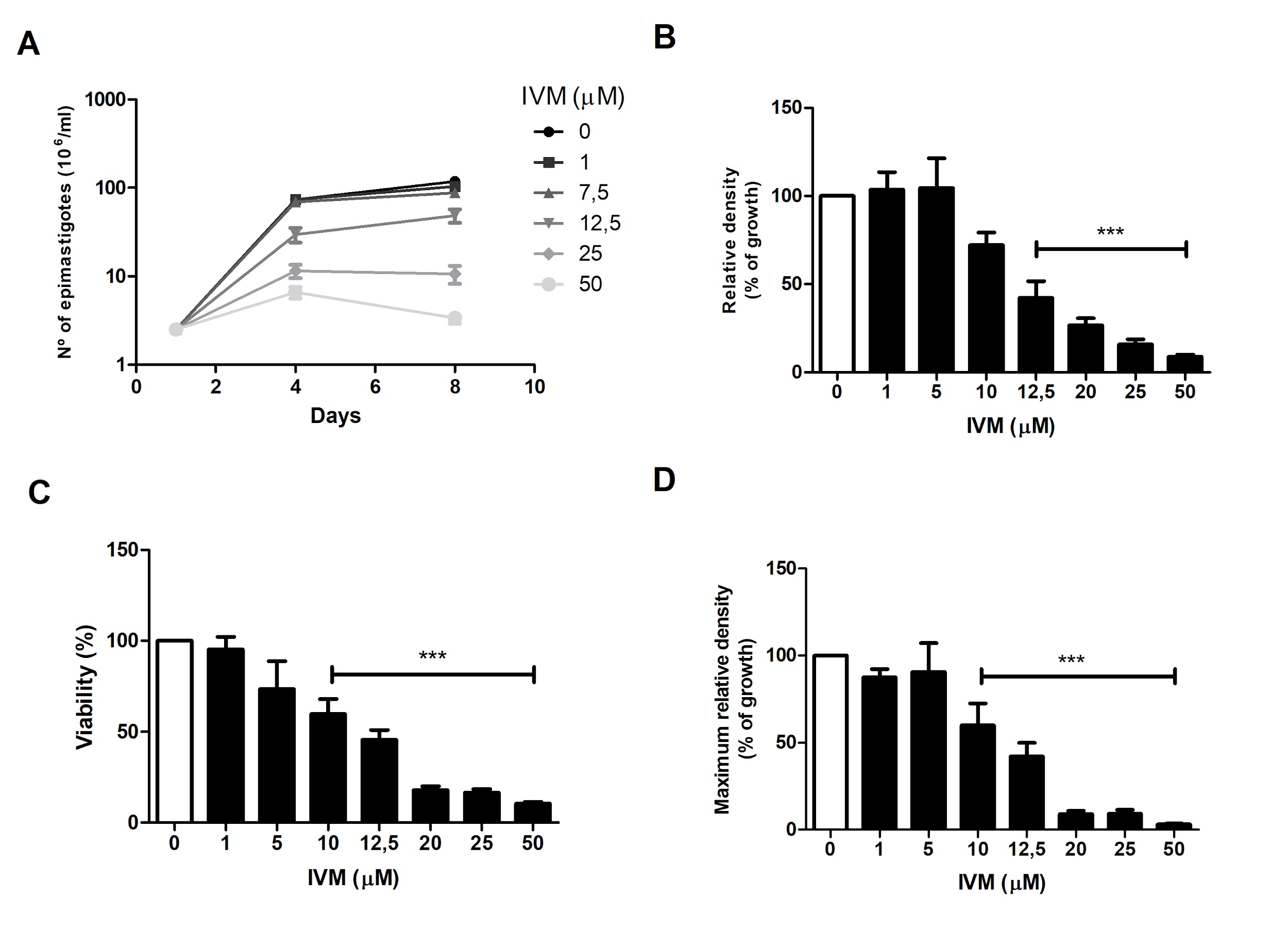


**Supplementary Figure 1.** *T. cruzi* epimastigotes (Y strain) were cultured in BHT media supplemented with 10% FBS during 8 days in the absence or presence of IVM. **(A)** Growth curves measured by counting in a hemocytometer chamber. The proliferation of epimastigotes was monitored on day 1, 4 and 8 of culture; **(B)** Relative density at day 4 of culture (related to control culture without IVM); **(C)** Viability percentage at day 4 of culture evaluated by MTT assay and **(D)** Relative maximum density calculated at day 8 of culture with counting data. ***p<0.001 ANOVA - Dunnett Test.


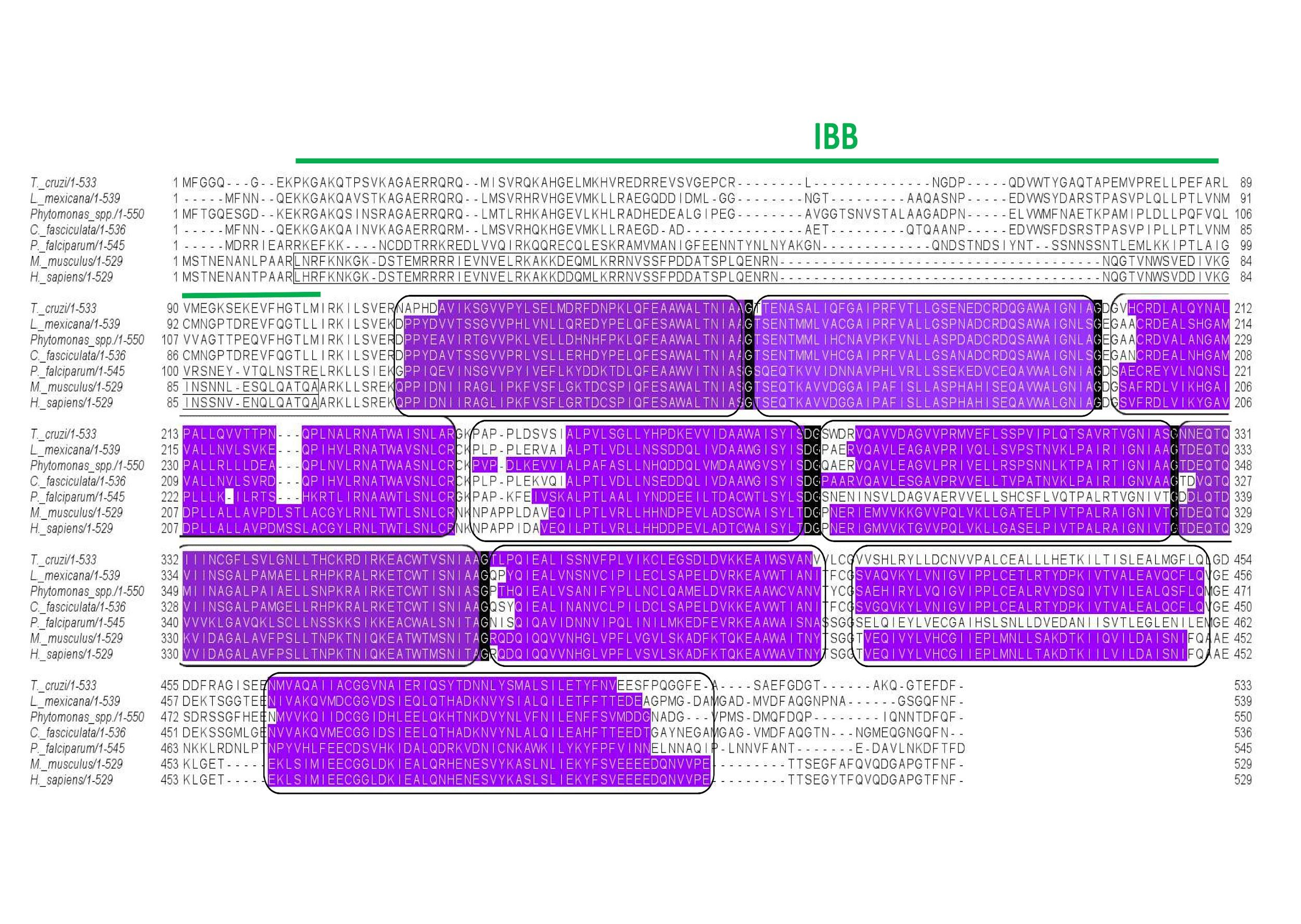


**Supplementary Figure 2.** **Multiple alignment of IMPα sequences of trypanosomatids and other eukaryotes and its structural analysis.** IMPα sequences of *T. cruzi*, *L mexicana*, *Phytomonas spp.* and *C. fasciculata*, together with those of *P. falciparum*, *M. musculus* and *H. sapiens* were analyzed with PFAM and SMART softwares. 7-9 ARM domains (purple), divided by a conserved Gly, are present in all the sequences evaluated. However, IBB domain (green) formed by the first 100-110 amino acids is only observed in mammals while absent in unicellular eukaryotes.
